# A Review of Detection Methods for Vancomycin-Resistant *Enterococci* (VRE) Genes: From Conventional Approaches to Potentially Electrochemical DNA Biosensors

**DOI:** 10.3390/bios13020294

**Published:** 2023-02-18

**Authors:** Nor Dyana Zakaria, Hairul Hisham Hamzah, Ibrahim Luqman Salih, Venugopal Balakrishnan, Khairunisak Abdul Razak

**Affiliations:** 1 Nanobiotechnology Research and Innovation (NanoBRI), Institute for Research in Molecular Medicine, Universiti Sains Malaysia, Gelugor 11800, Penang, Malaysia; 2 School of Chemical Sciences, Universiti Sains Malaysia, Gelugor 11800, Penang, Malaysia; 3 School of Materials and Mineral Resources Engineering, Universiti Sains Malaysia, Nibong Tebal 14300, Penang, Malaysia

**Keywords:** vancomycin-resistant *Enterococci* (VRE), DNA biosensor, electrochemical biosensor, Gram-positive bacteria

## Abstract

Vancomycin-resistant *Enterococci* (VRE) genes are bacteria strains generated from Gram-positive bacteria and resistant to one of the glycopeptides antibiotics, commonly, vancomycin. VRE genes have been identified worldwide and exhibit considerable phenotypic and genotypic variations. There are six identified phenotypes of vancomycin-resistant genes: VanA, VanB, VanC, VanD, VanE, and VanG. The VanA and VanB strains are often found in the clinical laboratory because they are very resistant to vancomycin. VanA bacteria can pose significant issues for hospitalized patients due to their ability to spread to other Gram-positive infections, which changes their genetic material to increase their resistance to the antibiotics used during treatment. This review summarizes the established methods for detecting VRE strains utilizing traditional, immunoassay, and molecular approaches and then focuses on potential electrochemical DNA biosensors to be developed. However, from the literature search, no information was reported on developing electrochemical biosensors for detecting VRE genes; only the electrochemical detection of vancomycin-susceptible bacteria was reported. Thus, strategies to create robust, selective, and miniaturized electrochemical DNA biosensor platforms to detect VRE genes are also discussed.

## 1. Vancomycin-Resistant *Enterococci* (VRE)

Since the late 1980s, vancomycin-resistant *Enterococci* (VRE) bacteria have been a primary nosocomial infection globally, and it is commonly spread via poor hospital hygiene practice. This contributes to higher treatment costs, more extended hospital stays, and a higher death rate [1]. VRE bacteria carry genes that are resistant to a wide variety of antibiotics such as beta-lactam, cephalosprin, trimethoprim-sulfametoxa, and glycopeptides (vancomycin and teicoplanin). Due to VRE being strongly resistant to a wide range of antibiotics, they could contribute to a major medical crisis. To overcome this, essentially, the last line of antibiotics used should mainly be a glycopeptide, such as vancomycin [2]. Conceptually, vancomycin impairs the growth of the bacteria’s cell wall, and resistance is based on altered targets for antibiotic–drug interactions. *Enterococci* that are vancomycin-susceptible synthesize cell wall precursors ending in D-Ala-D-Ala, which have a high affinity for vancomycin. Once bound, these precursors cannot participate in cell wall production. Thus, the bacteria are killed by the vancomycin. In the case of resistance, when vancomycin is introduced into the cell, VRE pathogens create precursors with alternative termini, such as D-Ala-D-Lac, that have a low affinity for bonding with vancomycin and can be employed to synthesize cell walls [1,3]. Vancomycin resistance towards the VRE family is classified into six distinct phenotypes: the VanA, VanB, VanC, VanD, VanE and VanG strains [3,4]. The highest level in the mechanism of resistance to vancomycin by VRE is caused by the presence of the VRE-VanA gene [5]. It is also resistant to teicoplanin.

**Figure 1:**
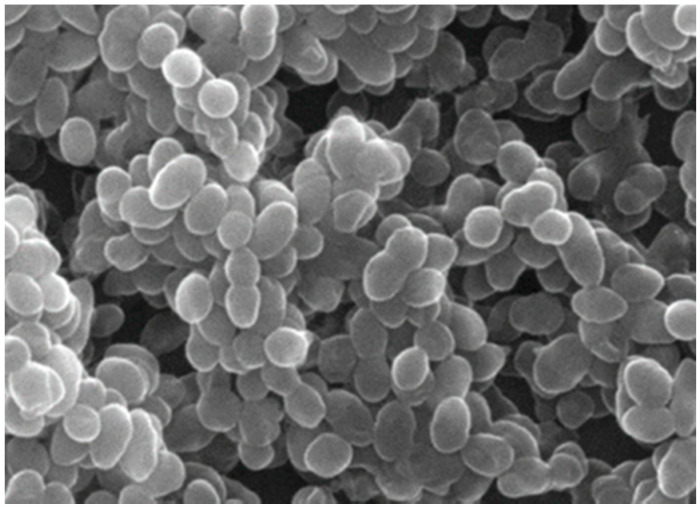
Scanning electron microscopy (SEM) of *Enterococcus faecalis* VRE cells, reproduced from ref. [7].

Meanwhile, the VRE-VanB gene is less frequently associated with resistance, as it can be treated with teicoplanin. In contrast to transposons-based resistance mechanisms, Van C resistance is chromosomal and non-transmissible to other bacteria and occurs naturally in Enterococci that are considered to be less virulent than *Enterococcus faecium* and *Enterococcus faecalis*, such *as Enterococcus gallinarum* and *Enterococcus casseliflavus*. Resistance to VanD and VanE has been reported sporadically [1,6]. Figure 1 shows a SEM image of *Enterococcus faecalis* VanB cells.

VRE can survive unharmed in the human bowel. When VRE strains increase significantly, they enter the bloodstream or other parts of the body via the proteolyzing chemicals they produce. VRE can also cause complications due to various illnesses, including bloodstream infections (sepsis), wound infections, urine infections, heart and brain infections, and pneumonia [1,8]. VRE infections are most frequently contracted by patients who are already ill in the hospital. These infections might be difficult to treat due to the limited number of effective antibiotics against the resistant bacteria. Certain VRE infections can be deadly. VanA and VanB have been commonly discovered in clinical laboratories, and the VanA strain is frequently identified due to its high level of vancomycin resistance [9,10].

Additionally, VanA strains can provide considerable complications for hospitalized patients because they can transfer to other Gram-positive infections, modifying their genetic material to increase their resistance to the antibiotics used during treatment. For instance, they may be able to transfer their resistance to other bacteria, such as MRSA (methicillin-resistant *Staphylococcus aureus*). These changed strains are now called *vancomycin-resistant Staphylococcus aureus* (VRSA) [11]. In terms of detecting VRE at the hospital, VRE can be diagnosed by a few standard methods such as antibiotic susceptibility, molecular diagnosis assays, and polymer chain reaction (PCR) using human samples of blood, urine, pus, or other fluid from the infected area [12].

### Detection of VRE

In the case of VRE detection, there are a few standard methods to detect VRE, such as traditional, immunoassay, and molecular methods used in clinical labs. The conventional techniques, for example, minimum inhibitory concentration (MIC) in broth or agar, disk diffusion, and E-test, are primarily based on the culture of these microorganisms in specific ways [8,13]. Even though the protocols are simple and easy to perform, they are not always valuable. A problem for some researchers is that some microorganisms are not easy to grow or have to grow for a long time before they are ready to be used. By contrast, there are a few different types of molecular methods, such as PCR [5], real-time PCR [14], and multiplex PCR [15]. However, they involve the amplification of the target genes. Because of their higher sensitivity and specificity for detection and quantification methods than traditional methods, they are essential tools for studying many different microbes. In addition, each technique has challenges, such as a long execution time and low reproducibility of results, and is also more expensive due to requiring expensive equipment and reagents. The RNA molecule is also unstable and needs specific equipment and bioinformatics skills to be used properly. Additionally, because both live and dead bacteria are found in the sample, the methods above can give high false-positive results. Another method to detect VRE is immunoassays, and the most commonly used are enzyme-linked immunosorbent assays (ELISA) and lateral flow immunoassays. ELISA is a labelled immunoassay that is called the gold standard of immunoassays and is probably the most widely used technology for clinical diagnosis. It is based on antigen-antibody reactions where an enzyme labelled on a secondary antibody is needed. However, this approach is costly due to antibody and enzyme preparations as well as the process being laborious. In addition, the most common problem of ELISA is antibody instability.

In recent years, biosensors have emerged as a superior technique for assisting PCR and ELISA in detecting biomolecule analytes such as cancer biomarkers, viruses, and bacteria. Thus, biosensors can be used in addition to PCR and ELISA to identify and quantify bacteria [16]. According to the International Union of Pure and Applied Chemistry, a biosensor must have a biorecognition element such as an enzyme, DNA, or antibody in direct contact with a transduction element to work [17]. In terms of transduction elements, a variety of transducers can be used to develop electrochemical, optical, thermal, or piezoelectric biosensors. In addition, a biosensor should be able to provide quantitative or semi-quantitative analytical information and measurement without the need for extra steps or reagents.

Among the many developed biosensors, electrochemical biosensors are commonly developed during clinical diagnosis due to the rapid response time, high sensitivity, high selectivity, and less expensive instrumentation, and the detection systems are easy to miniaturize. Hence, this review discusses the strategies for detecting VRE based on conventional, immunoassay, molecular, and electrochemical DNA biosensor methods. However, in the literature search, no work on detecting VRE genes using electrochemical techniques has been published. Most of the work is reported based on the electrochemical detection of vancomycin compounds. Thus, we discuss the published articles that detect VRE strains with various approaches and the strategies for developing electrochemical DNA biosensing of VRE genes.

## 2. Standard Clinical Diagnosis for VRE

### 2.1. Traditional Methods

**Figure 2:**
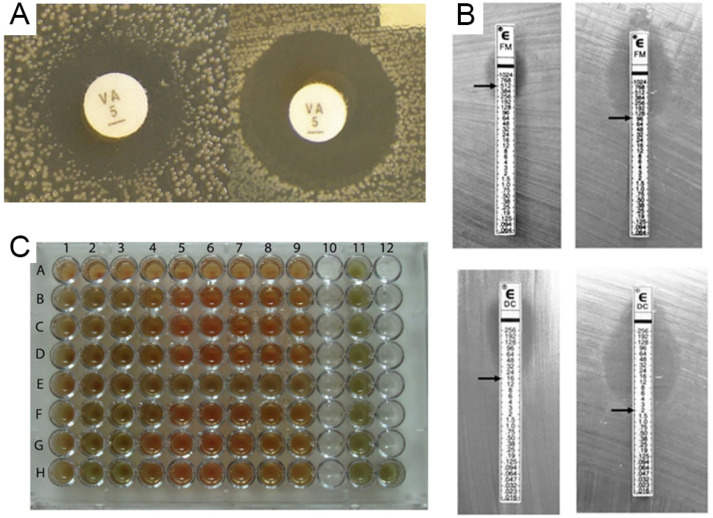
Representation of various conventional antibiotic susceptibility testing methods. (**A**) Disk diffusion, demonstrating inhibition zones, adapted from [21]. (**B**) Example of a GRD Etest used to detect vancomycin-intermediate *Staphylococcus aureus*, adapted from [22], (**C**) Illustration of an example of the 96-well micro plate’s broth microdilution method for two *Streptococcus pneumoniae* strains’ vancomycin assays, reproduced from Ataee and co-workers [23]. In that plate, the 9 wells in row A serve as a negative control, while rows B, C, and D for strain one and rows F, G, and H for strain two contain vancomycin concentrations ranging from 0.5 to 128 g/well for the MIC determination. Nine wells in the E row and 11 columns are designated as positive controls.

Traditional methods such as disc diffusion [18], minimum inhibitory concentration (MIC), and broth dilution for the detection of VRE are regularly used in clinical labs to identify and quantify bacteria (as shown in Figure 2). They are based on the colonies’ morphological and biochemical properties, making them simple in counting the cultivable bacteria present in the medium. They are widely used for identifying specific bacteria due to their high sensitivity [19,20].

The standard procedure for routine antibiotic susceptibility testing in many clinical microbiology laboratories is agar disk-diffusion testing, established in 1940 [24]. The Clinical and Laboratory Standards Institute (CLSI) now provides and recognizes the standards for testing bacteria and yeasts. The agar disc diffusion method involves the diffusion of VRE over agar via an antimicrobial impregnated in a paper disc, where the disc inhibits the microbial growth circle [25]. It is a qualitative technique that assigns a sample to three categories: resistant, intermediate, or susceptible. Additionally, it is a practical, low-cost, simple procedure perfect for germs that multiply quickly. However, significant limitations exist, including using VRE that do not diffuse well in agar [24]. Ranjit Sah et al. reported the first case of VRE causing diarrhea in a kidney transplant patient in Nepal. Minimum inhibitory concentration (MIC) testing using the agar dilution method validated the isolate’s resistance to vancomycin, showing that the MIC was higher than 16 mcg/mL [26].

The most broadly used approach for measuring VRE susceptibility is the MIC. This method determines the smallest quantity of antibiotic required to stop bacterial growth on agar, broth plates, or both and inoculate agar and broth with a certain number of bacteria before experimenting (usually 0.5 in the MacFarland standard). Microbiological growth is then monitored after incubation. This is a low-cost approach that does not necessitate the purchase of special equipment. Once the minimum inhibitory concentration (MIC) of an antibiotic has been determined, the therapeutic concentration of the antibiotic can be adjusted. The presence of resistance in non-cultivable VRE cannot be determined. The experiment’s effectiveness depends on the incubation period, the diluted antibiotic concentration, and the quantity of VRE implanted. As a whole, it is a semi-quantitative approach that cannot accurately identify MICs [20,24].

In another method, the E-test is a combination of the two previous procedures. E-test follows a disc diffusion-like approach; nevertheless, it determines the MIC. On an agar plate, a rectangular gadget is inserted using the antimicrobial concentration gradient and the interpretation scale is used on the other. While the E-test^®^ has the same limitations as the previous two tests (i.e., execution time), it features an immobilized VRE gradient indicated on the ruler, which allows for a more straightforward method of directly quantifying the susceptibility of microorganisms, particularly those that are difficult to culture (e.g., *Haemophilus influenzae* and *Mycobacterium bovis*) or even anaerobes [24]. Geetarani et al. investigated the phenotypic and genotypic characteristics of VRE from infections with clinical significance in hospitalized patients and how colonization of the gut is related to these infections. The E-test was used to determine the MIC for teicoplanin, linezolid, tigecycline, daptomycin, and quinupristin-dalfopristin [12].

Another method for detecting VRE is broth dilution. It is one of the most basic VRE-testing methods. The general approach of the broth dilution method involves preparing two-fold dilutions of the antimicrobial agent (e.g., 1, 2, 4, 8, 16, and 32 mg/mL) in a liquid growth medium dispensed in the tube containing a broth microdilution (BMD) or with smaller volumes using a 96-well microtitration plate (microdilution). Then, each line or well is inoculated with a microbial inoculum and prepared in the same medium after diluting a standardized microbial suspension. The reliable and well-standardized broth microdilution method is beneficial in research investigations and when testing a single antimicrobial agent for a single bacterial strain. Beniamino et al. developed two screening assays to detect VRE. The microdilution broth test was conducted in accordance with CLSI recommendations. The minimal inhibitory concentration (MIC) for all strains was determined to be 4 g/mL, as the wells with this concentration exhibited no observable growth at first [27]. Although convenient microdilution equipment is readily available on the market and the approach is labor- and time-intensive, it is typically not practical for routine usage in clinical microbiology laboratories.

### 2.2. Immunoassay Methods

**Figure 3:**
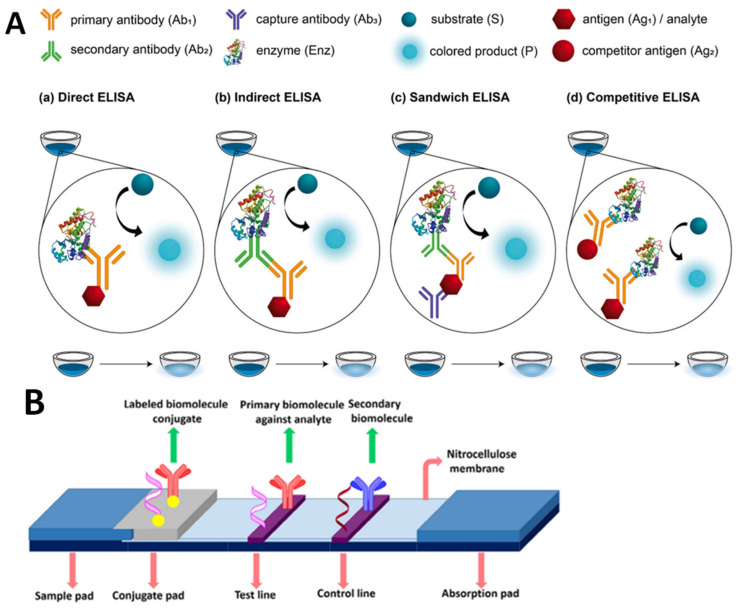
Schematic illustration of an immunoassay method. (**A**) Various types of ELISA (adapted from Ref. [29]). (**B**) The basic structure of lateral flow assay, reproduced from Bahadir and Sezgintürk [30].

Immunoassays are regularly used to analyze proteins, hormones, viruses, microbes, DNA sequences, and medicines in the therapeutic, environmental, agricultural/food, and forensic industries [28]. The enzyme-linked immunosorbent assay (ELISA) is a labeled immunoassay called the gold standard of immunoassays. It is probably the most widely used technology for quantitative screening of high-throughput samples, as shown in Figure 3A.

The free analyte and an enzyme-labeled compound compete for binding toward immobilized antibodies in this format. The enzyme label indicates the displacement caused by a colorimetric reaction, which is amplified by the numerous rotations of the enzymatic reaction. In comparison to the ELISA, a lateral-flow immunoassay (LFIA) strip is quicker and more straightforward. LFIA strips, as an instrument-free approach, can be utilized for on-site qualitative. The critical aspect of a lateral flow assay consists of four components: a sample pad, which is the area on which the sample is dropped; a conjugate pad, which is the location on which labeled tags are combined with biorecognition elements; a reaction membrane (typically nitrocellulose membrane), which is the location of the test line and control line for the target DNA-probe DNA hybridization or antigen–antibody interaction; and an absorbent pad, which is the location on which the waste is reserved. Figure 3B provides an illustration of the lateral flow assay’s fundamental structure. Dezhao et al. [31] designed an indirect competitive ELISA (ic-ELISA) and lateral-flow immunochromatographic assay (ICA). They tested in raw milk and animal-feed samples. Ic-ELISA recovery rates ranged from 89.2% to 121.60%. It was 5 ng/g for raw milk and 200 g/kg for animal feed samples in the lateral-flow ICA strip. Both approaches worked well in different samples. Ic-ELISA was sensitive and sufficient for high-throughput screening. The lateral-flow ICA strip is a quick and easy on-site diagnostic tool. Another example of the rapid LFIA method was successfully performed by Oueslati et al, in which VanA-producing VRE (VanA-VRE) were detected in colonies and broth. In less than 15 min, all 40 VanA-VRE clinical isolates were accurately identified regardless of the species expressing the VanA ligase and the medium utilized for bacterial growth. There was no cross-reactivity with any other therapeutically relevant ligases (VanB, C1, C2, D, E, G, L, M, and N), and it showed that the sensitivity and specificity of the assay were 100% for VRE-VanA grown on Mueller–Hinton agar plates. The LODs were 6.3 × 10^6^ cfu and 4.9 × 10^5^ cfu per test [32]. Bian et al. performed an LFIA by combining Europium (Eu) (III) chelate nanoparticles (CNEUs), which were able to perform real-time monitoring of VRE concentration in serum within 15 min. In this study, combining the fluorescence-based LFIAs with portable strip readers fulfills the criteria for semiquantitative detection. Commercial (Eu (III)) chelate-dyed nanoparticles with modified carboxylic acid groups can encapsulate thousands of fluorescent chelates in a single polystyrene shell, resulting in improved labeling strength and high lanthanide-specific fluorescence. The advantages of using immunoassay markers for trace analysis include their long fluorescence decay time, large Stoke shift between excitation light and emission light, narrow excited fluorescence band, sharp fluorescence emission, and excellent photo-stability, all of which result in exceptional analysis sensitivity and precision. As a result, the CN-EU-based LFIA assay has a detection limit of 69.2 ng·mL^−1^ and a wide linear range of 0.1–80 g·mL^−1^ for quantifying VAN concentration [33]. Odekerken et al. developed an easy-to-use ELISA method capable of detecting gentamicin and vancomycin in protein-containing samples such as serum and wound exudate. For vancomycin, the ELISA is accurate between 20 and 5000 ng/mL without any crossreactivity with gentamicin, which mean the sensitivity for both ELISAs is very high [34].

Even though the LFIAs are possibly the most cost-effective, time-efficient, and user-friendly paper-based point-of-care tests, there are limitations in the diagnostic field, including poorer sensitivity (more false negatives) and lower specificity (more false positives) than laboratory tests. In addition, this approach has a number of limitations, including reagent stability, the need for chilled shipping and storage, batch-to-batch (or clone-to-clone) variability, and high cost of producing antibodies.

### 2.3. Molecular Methods

**Figure 4:**
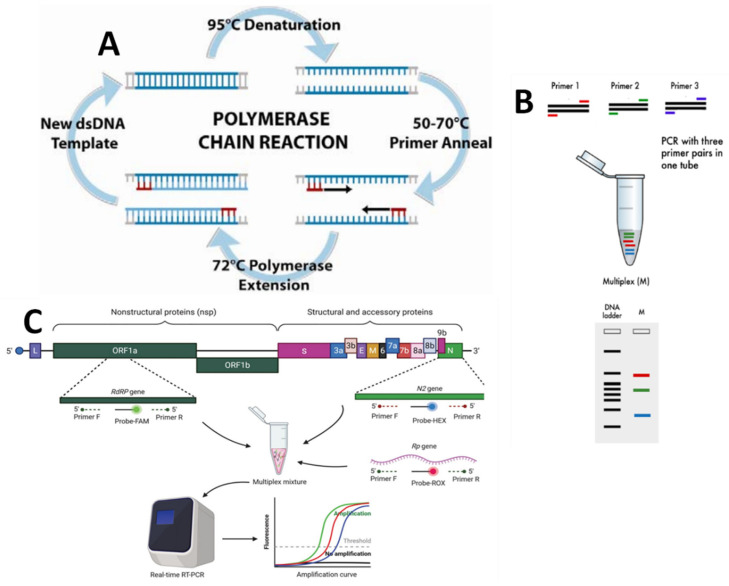
Schematic illustration of a molecular detection method: (**A**) the polymerase chain reaction (PCR) amplification process (adapted from Ref. [35]); (**B**) multiplex PCR (reproduced from Ref. [36]); (**C**) reverse transcriptase PCR (reproduced from Ref. [37]).

Traditional methods of identification, which rely on growing bacteria, are time-consuming and insufficiently selective. Disk diffusion testing methods have been demonstrated to be less sensitive than broth dilution methods in detecting VRE strains. On the other hand, the broth methods require a 24 h incubation period to detect vancomycin-resistant bacteria. It is critical to identify VRE-colonized patients quickly so that adequate control measures can be implemented to prevent the virus from spreading. Alternative methods for detecting and identifying VRE include molecular methods such as PCR-based approaches. PCR is an in vitro method for exponentially amplifying specific DNA and RNA sequences, as displayed in Figure 4.

The theory of PCR is based on the isolation, amplification, and quantification of a specific DNA sequence present in the genetic material of the target microorganism. It is a commonly used technology due to its excellent specificity. In the laboratory, PCR is used to quickly detect bacteria from a variety of settings, as well as resistance genes. One of its main benefits is that it may amplify existing genes from non-growing or dead microorganisms, making them accessible for discovery in contrast to traditional methods. Traditional methods frequently produce erroneous (e.g., false negative) interpretations based simply on phenotypic traits, which can be avoided when standard PCR techniques are used. Paule et al. [36] demonstrated that the specific multi-plex PCR assay using VanA and VanB primers was more sensitive than culture on selective media for the detection of gastrointestinal colonization by VRE in samples collected by rectal swabs (20 of 46 versus 8 of 46; *p* = 0.001) and perianal swabs (17 of 58 versus 12 of 58; *p* = 0.059). Sevim zsoy et al. [33] investigated the prevalence of VRE in stools submitted for a C. diff. toxin assay and the correlation between C. diff. and VRE. Vancomycin and teicoplanin minimum inhibitory concentrations (MICs) were determined using the E-test, and VanA and VanB, molecular resistance genes, were identified using PCR after species identification. With the PCR method, Amberpet et al. analyzed the risk factors associated with VRE colonization. They calculated the prevalence of colonization in patients admitted to the Medical Intensive Care Unit (MICU). PCR tested all phenotypically vancomycin-resistant isolates for vancomycin resistance genes (VanA, VanB, VanC1, and Van C2/C3) with published primers, as shown in Table 1 [38].

A multiplex reaction, an upgraded method of conventional PCR, can be used to optimize the PCR. In this procedure, some primers are utilized in the solution mix so that more than one microbe can be identified and differentiated in a single run. The great advantage is the reduced cost and time by amplifying different genes at the same time. For example, Lu et al. [39] have developed a single-tube, multiplex PCR assay to detect VRE and to identify relevant vancomycin-resistant genotypes. The Detection of VRE from the broth of cultured nosocomial surveillance specimens was also compared to traditional culture methods for sensitivity and specificity. The multiplex PCR has a sensitivity of 97.9% and a specificity of 100% compared to the standard culture approach for detecting and identifying VRE genes in Enterococci directly from the culture-positive broth. For the detection of eight different Enterococcus species (*E. faecalis*, *E. faecium*, *E. gallinarum*, *E. casseliflavus*/*E. flavescens*, *E. raffinosus*, *E. avium*, *E. hirae*, and *E. durans*), Takahiro Nomura et al. [40] investigated new colony multiplex PCR methods. These assays demonstrated significant sensitivity and specificity for detecting van-comycin resistance determinants and *Enterococcus* spp. in 135 enterococcal isolates used in this study. Vancomycin re-susceptibility among enterococci isolates was also investigated in a study by Bhatt et al. [15]. The percentage of isolates resistant to vancomycin by vancomycin E-test (MIC 32 mg/mL) was 14.6% (14/96), which is high. Thirteen of the fourteen isolates showed resistance to teicoplanin at the 16-microgram-per-milliliter level. All 14 isolates were tested, and the VanA gene was found. Table 1 shows that PCR primers and DNA probes are used to detect VRE genes.

**Table 1:** PCR primers and DNA probes used for the detection of VRE genes.

Primer or Probe	Sequence (5′ > 3′) a	Size of Sequence (bp’s)	Amplified Gene or DNA Target Sequence	Ref
VanA (+)	GGGAAAACGACAATTGC	732	VanA VanA	[15]
VanA (−)	GTACAATGCGGCCGTTA			
VanB (+)	ACGGAATGGGAAGCCGA	647	VanB	
VanB (−)	TGCACCCGATTTCGTTC			
VanC	ATGGATTGGTAYTKGTATc		Van C1/2	
VanC	TAGCGGGAGTGMCYMGTAAc			
VanD	TGTGGGATGCGATATTCAA	500	VanD	
VanD	TGCAGCCAAGTATCCGGTAA			
VanE	TGTGGGATCGGAGCTGCAG	430	VanE	
VanE	ATAGTTTAGCTGGTAAC			
VanG	CGGCATCCGCTGTTTTTGA	941	VanG	
VanG	GAACGATAGACCAATGCCTT			
VanA	5′-CATGAATAGAATAA AAGTTGCAATA-3′	1032	VanA	[41]
	5′-CCCCTTTAACGCTA ATACGACGATCAA-3′			
VanA1, VanA2	5′-GGGAAAACGACAATTGC 3′ and 5′-GTACAATGCGGCCGTTA 3′	732 bp	VanA	[42]
VanB1, VanB2	5′-ATGGGAAGCCGATAGTC-3′ and 5′-GATTTCGTTCCTCGACC-3′	635 bp	VanB	
VanA	GCT ATTCAG CTG TAC TC CAG CGG CCA TCA TAC GG	783 bp	VanA	[18]
VanB	CAT CGC CGT CCC CGA ATT TCA AA GAT GCG GAA GAT ACC GTC GCT	297 bp	VanB	

The RNA molecule is reverse-transcribed into a complementary DNA molecule in a reverse transcriptase polymerase chain reaction (RT-PCR). Following this step, amplification is carried out using conventional PCR. It is a highly specific, sensitive, and reliable approach. The cDNA molecule produced from the initial RNA has been proven to be purer than DNA extracted directly from the target. A conventional DNA molecule contains contaminants such as proteins absent from cDNA. As a result, cDNA is more specific and easier to detect using primers.

Additionally, the technique enables the identification of replicating cells with high sensitivity, allowing for the differentiation of living from dead bacteria. Moreover, RT-PCR is utilized to analyze gene expression qualitatively and is required for other molecular techniques such as qPCR for quantifying RNA levels and microarray for detecting multiple target gene expressions. On the other hand, the high instability of the RNA molecule is a significant disadvantage of this approach. As a result, sample processing is the most difficult and takes a lot of time and effort, which makes analysis time-consuming and costly. The use of RT-PCR to detect VRE genes is not so widely described in the literature. A combination of primary culture on chromogenic media and rapid confirmation of suspect colonies by PCR can provide improved specificity and faster reporting times [43].

With the use of standard primers, Zerrouki et al. [14] have established a robust and sensitive RT-PCR test. The system was created online with Primer3. In silico PCR and BlastN analyses were used to test the primer and probe specificity. Using genomic DNA from 255 bacterial isolates, including *Enterococcus* spp., Gram-positive and Gram-negative strains, and 50 stool and rectal swab samples, the specificity of the new real-time PCR (RT-PCR) approach was determined. From all evaluated vancomycin-resistant isolates carrying the VanA or VanB genes, the generated RT-PCR was 100 percent specific and positive. The RT-PCR detection limits for the VanA and VanB genes were found to be 47 and 32 CFU/mL, respectively.

Effective vancomycin resistance identification is critical to preventing nosocomial infections and outbreaks. Although the molecular detection method is widely regarded as an excellent option for detecting VRE, it has a few drawbacks that can be problematic. It can be time-consuming and requires expensive reagents and equipment (such as a thermal cycler, gel electrophoresis, etc.).

## 3. Potential and Strategies in Electrochemical DNA Biosensors of VRE Genes

As we have already discussed in Section 1, electrochemical biosensors are nowadays commonly developed in electroanalytical research. The basic principle of an electrochemical DNA biosensor is monitoring electrochemical signals after the formation of hybridized dsDNA on the electrode surface. This can happen when the immobilized ssDNA probe sequence can capture its complementary ssDNA (target) to form double-stranded nucleic acid (dsDNA) film at the modified electrodes. The dsDNA formation can then be commonly monitored through the generated current (*i*) utilizing voltammetric measurements or charge transfer resistance (*R*ct), measured using electrochemical impedance spectroscopy (EIS) either via label-free or labeled detection systems. In Section 3.3, the features of label-free and labeled systems are addressed.

Electrochemical DNA biosensors are the ideal solution for detecting VRE genes because the electrochemical DNA platform-based immobilized ssDNA probe on the electrode surface may be specifically designed to detect a range of VRE genes. Furthermore, electrochemical DNA biosensors have a variety of excellent characteristics, such as simplicity, ease of use, low cost, high sensitivity, high selectivity, rapid response, portability, and multiplexing capability, which make them widely applicable as clinical and point-of-care disease diagnostics. As previously described in earlier sections, there are a number of clinical laboratory assays available to detect VRE genes, the most popular of which is the PCR method. Although PCR is commonly used in many hospitals to detect VRE genes, it is quite expensive because it requires specific machines, complex processes, long analysis time, and an experienced worker to perform this test.

**Figure 5:**
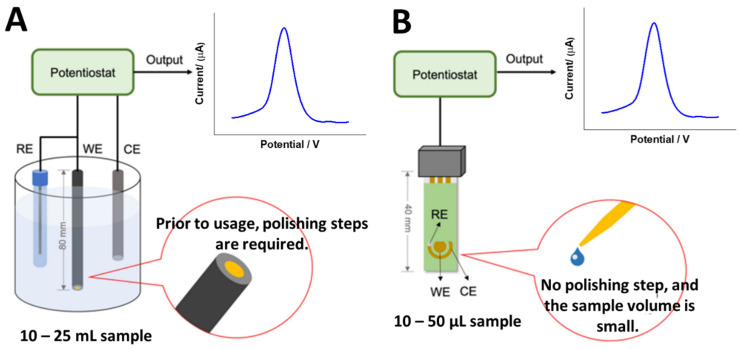
Types of electrode systems that can be used to develop electrochemical DNA biosensors to detect VRE genes. (**A**) Conventional electrodes and (**B**) screen-printed electrodes (miniaturized electrodes). This figure has been reproduced from Awang et al. [45], under Attribution 4.0 International (CC BY 4.0) License.

To the best of our knowledge, no study has yet been published on the use of electrochemical DNA biosensors for detecting VRE genes. This is thought to be due to the fact that there are six varieties of VRE strains: VanA, VanB, VanC, VanD, VanE and VanG. Therefore, creating electrochemical DNA biosensor platforms for detecting VRE genes will be a very novel effort for the researchers. Nevertheless, according to the recent literature, the simplest method is to create a detection system based on immobilized vancomycin molecules on the electrode surface for detecting the whole bacteria, as reported by Norouz et al. [44] Despite its simplicity, the detection approach is not that selective in detecting certain VRE genes, as this approach cannot distinguish the specific VRE strains. Hence, to develop electrochemical DNA biosensors, single-stranded DNA (ssDNA) will be immobilized on the electrode surface using various immobilization methods such as physical adsorption, self-assembly monolayer (SAM), covalent immobilization, and a few more. Nevertheless, the most common method used to obtain monolayer and uniform immobilized ssDNA layers are self-assembly monolayer and covalent immobilization. In terms of electrodes, the typical type of electrode materials used are glassy carbon (GC), gold (Au), and platinum (Pt) electrodes. Meanwhile, the miniaturized electrodes are screen-printed carbon electrodes (SPCEs) and screen-printed gold electrodes (SPGEs), as shown in Figure 5. Hence, the potential and strategies for developing electrochemical DNA biosensors to detect VRE genes are discussed.

**Figure 6:**
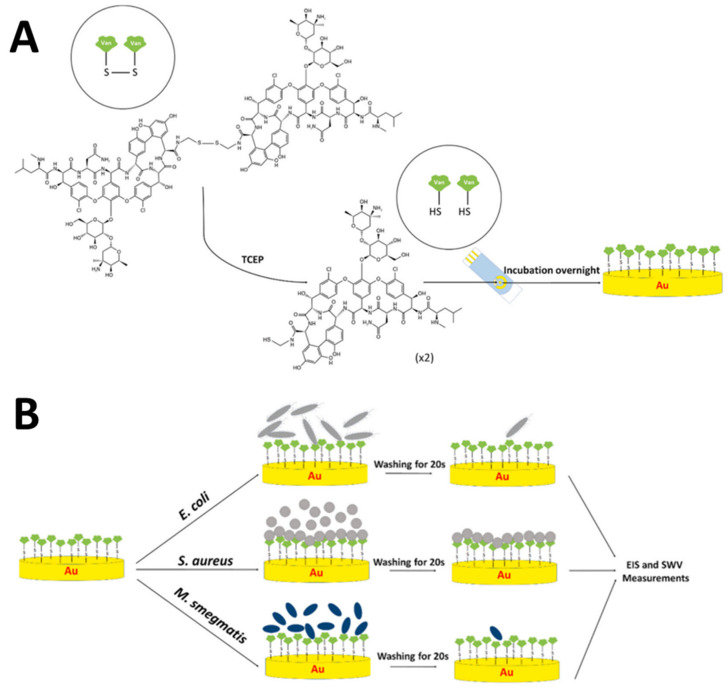
Schematic of Gram-positive bacteria detection based on a self-assembled monolayer (SAM) of vancomycin molecules on screen-printed electrode gold surfaces. (**A**) Synthesis of Bis-vancomycin (Bis-Van) molecules with a disulfide bond and immobilization of Van molecules onto the SPGE surface via thiol chemistry. (**B**) Interaction of Gram-positive bacteria with Van-modified SPGEs. This figure has been retrieved from Dizaji and co-workers [44].

From the literature search, no report so far reported the detection of VRE genes utilizing electrochemical DNA biosensors. Nonetheless, only one fascinating paper has been reported on the detection of Gram-positive-bacterial using an immobilized vancomycin (Van) molecule on the SPGE surface to create an antibiotic-based biosensor platform [44]. In their work, Van molecules were first functionalized with thiol groups using cystamine dihyrochloride to obtain Bis-Van molecules in which the Bis-Van has two vancomycin molecules conjugated together via a disulfide bond, as displayed in Figure 6A. Subsequently, the disulfide bond was broken using Tris(2-carboxyethyl) phosphine hydrochloride (TCEP) to obtain a single thiolated-Van. As-synthesized thiolated-Van was obtained, and HS-Van molecules were immobilized onto the SPGE surface for 18–24 h, as illustrated in Figure 6B. Atomic force microscopy (AFM), scanning electron microscopy (SEM), cyclic voltammetric (CV), and electrochemical impedance spectroscopic (EIS) measurements were carried out to characterize the Van-modified SPGEs. To test the selectivity of the modified electrodes towards Gram-positive bacteria, three different types of bacteria were used, *E. coli* and *M. smegmatis* as vancomycin-resistant models and *S. aureus* as a vancomycin-susceptible model. Based on the EIS responses, *S. aureus* was strongly attached to the immobilized Van molecular probe, whereas *E. coli* and *M. smegmatis* did not show any binding properties on the electrode surface. For the analytical calibration curve, different concentrations of *S. aureus*, ranging from 10 to 108 CFU/mL were measured, and the LOD was found to be 10.158 CFU/mL. Thus, their results concluded that a novel antibiosensor platform has been successfully developed and can potentially be used as a sensor platform for detecting vancomycin-susceptible bacteria.

As no paper has yet reported on the electrochemical DNA biosensor platform for the detection of VRE genes, we thus propose several essential strategies for sensing VRE strains. Before detecting the VRE genes, the immobilized single-stranded DNA (ssDNA) molecular probe layer must be created on the electrode surface. It is not only the formation of the ssDNA layer but also, most importantly, the stability and uniformity of the immobilized ssDNA chains on the electrode surface that must be considered. To obtain a robust and well-immobilized ssDNA probe and highly oriented immobilized ssDNA chains that can increase the efficiency of hybridization reactions, as discussed previously, SAMs and covalent attachment can be employed. Although the physical adsorption is easy to prepare, the formation of the ssDNA probe is not strong enough to be retained on the electrode surface as electrostatic forces only form the immobilized DNA. Thus, physical adsorption is not discussed in this review.

### 3.1. Immobilization ssDNA Probe-Based Self-Assembly Monolayer (SAM)

**Figure 7:**
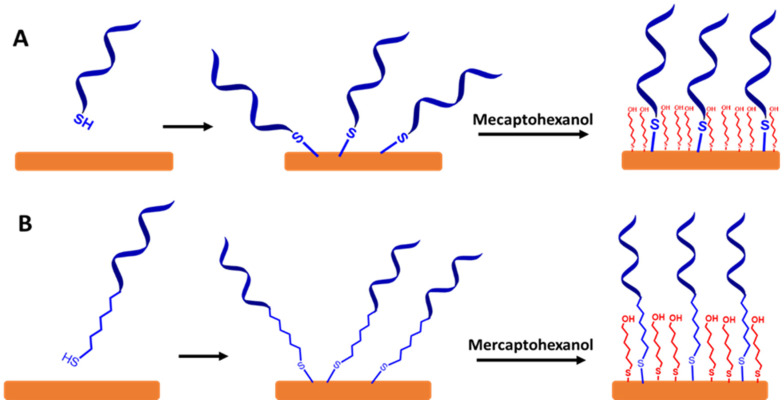
A schematic representation for a self-assembled monolayer of ssDNA probes on the gold electrode surfaces via thiol chemistry. It is followed by capping the surface with mercaptohexanol. (**A**) Self-assembled monolayer of functionalized thiol ssDNA chains and (**B**) self-assembled monolayer of functionalized thiol-alkyl ssDNA chains. Relative sizes of gold electrodes, ssDNA chains, alkyl ssDNA chains, and mercaptohexanol are not to scale.

To create a self-assembled monolayer ssDNA probe in sensing VRE genes, it is common for the ssDNA chains to be directly functionalized with a thiol group (SH) or functionalized thiol-alkyl ssDNA chains at 3′ or 5′ terminal as a sulfur head group. The best electrode material for these approaches is gold, as illustrated in Figure 7A,B, respectively. This is because the thiol groups can easily bind to the gold substrate to form the thiol-gold (S-Au) bond via strong chemisorption [46]. Moreover, the surface of gold does not require a stable surface oxide and does not undergo unusual reactions (e.g., corrosion) [47,48]. The couplings between the thiol group and the Au electrode surface involve van der Waals interactions, hydrophobic interactions, hydrogen bonds, and a semi-covalent binding between S and Au. The following step is capping the residual active surface with a backfilling agent such as mecaptohexanol to orientate the ssDNA chains in the upright position [49].

**Figure 8:**
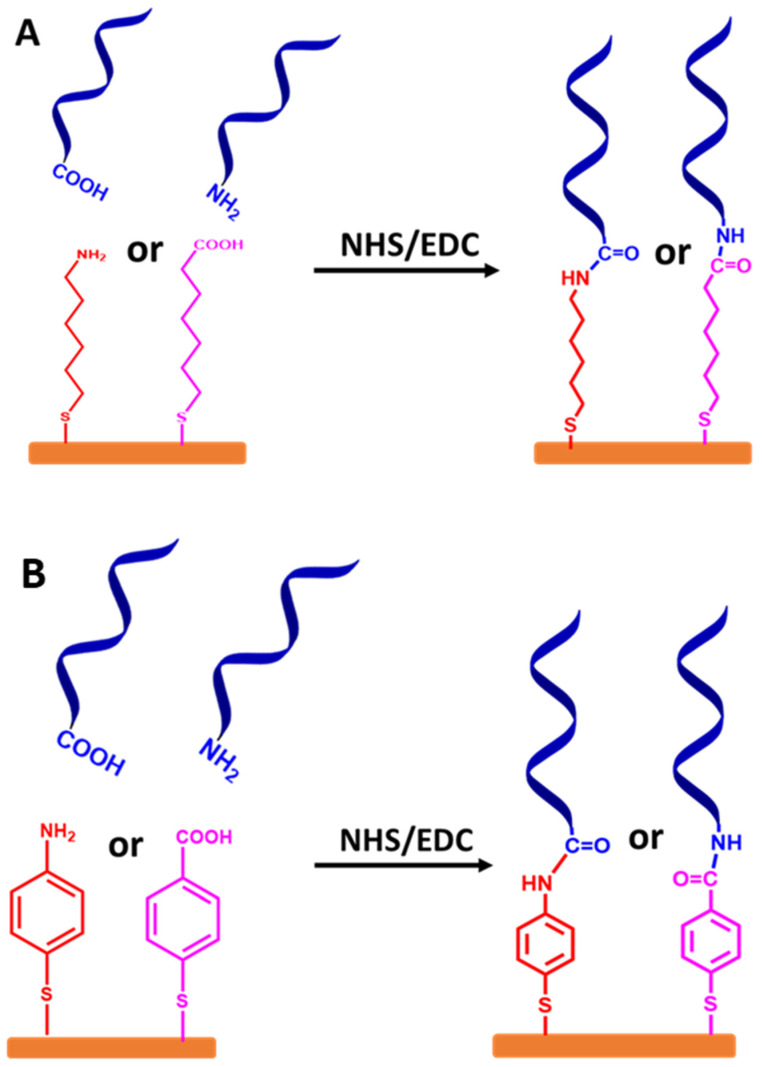
Schematic representation for stepwise protocols of self-assembled monolayer ssDNA chains onto the gold electrode surfaces via functionalized organic linkers with thiol groups, followed by coupling ssDNA chains to the linkers using NHS and EDC coupling reagents. (**A**) Self-assembled monolayer of ssDNA chains via thiolated alkyl linkers and (**B**) self-assembled monolayer of ssDNA chains via thiolated aromatic linkers. The relative sizes of gold electrodes, thiolated alkyl and thiolated aromatic linkers, and ssDNA chains are not to scale.

Stepwise protocols based on a spacer from an alkyl chain or aromatic ring are used in different approaches. The ssDNA chains are attached at the terminal end group (tail) via NHS and EDC chemistry, as demonstrated in Figure 8A,B, respectively.

**Figure 9:**
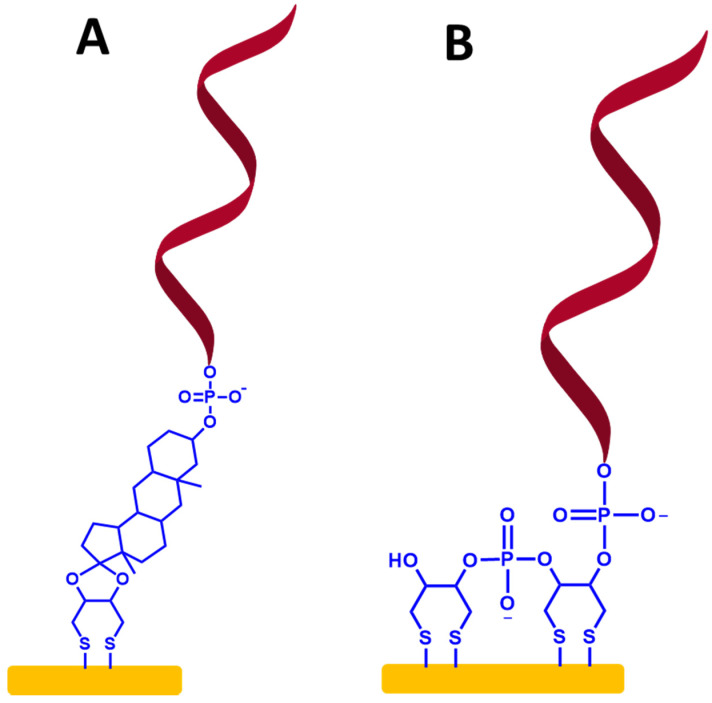
(**A**,**B**) Self-assembled monolayer ssDNA probes on the gold electrode surface-based dithiol groups. The relative sizes of gold electrodes, dithiol groups, and ssDNA chains are not to scale.

On the other hand, to make more stable immobilized ssDNA probes, previous studies showed that a SAM-based dithiol group has been used for the immobilization of ssDNA probes [50,51,52]. To create this type of ssDNA probe immobilization, the organosulfur molecules that have two identical thiol substituents, can provide two attachment points on the metallic surface, as shown in Figure 9A,B.

**Figure 10:**
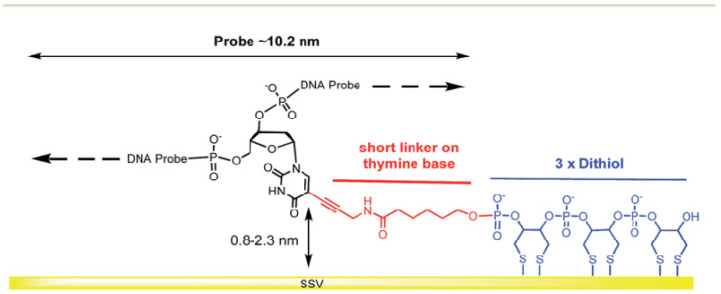
Self-assembled monolayer of 30-mer ssDNA probes on a gold sphere segment void (Au SSV) surface via three dithiols as a surface anchor that promotes horizontal orientation of dsDNA molecules. This figure has been adapted from Bartlett and his group [51], under Attribution 3.0 Unported (CC BY 3.0) License.

This makes more stable ssDNA probes than immobilized ssDNA probe-based monothiols because of a multivalent mechanism of interactions between the dithiol group and Au surface. Additionally, it provides sufficient distance for an immobilized ssDNA probe, enabling enhanced mobility and flexibility of the ssDNA chains. An exciting work has been demonstrated by Bartlett et al. [51], where 30-mer single-stranded DNA probes were self-assembled on the gold sphere segment void (Au SSV) surface by the three dithiols for monitoring the hybridization of ssDNA to double-stranded DNA (dsDNA) by electrochemically driven melting, as displayed in Figure 10. The primary purpose for using this form of connection at the Au SSV was to prevent ssDNA probe desorption at negative potentials.

Although chemisorption of the SAM-ssDNA on the gold electrode surfaces is simple to accomplish, it has been established that the resultant SAMs lack of stability since the Au-S bond energy is only around 43 kcal/mol. As a result, the bonds between Au and S become weak and are easily oxidized [48,53].

### 3.2. Immobilization ssDNA Probe-Based Covalent Attachment

To overcome the lower stability of the immobilized ssDNA probe-based SAMs, the covalent immobilization method is the best approach to obtaining more stable and robust ssDNA immobilization for sensing VRE strains. Before immobilizing ssDNA probes, functionalized organic linkers with amine, amide, or carboxylic acid groups are needed to be immobilized onto the electrode surfaces. Two main types of organic linkers that can be used are alkyl and aromatic ring linkers. The most suitable method to anchor the organic linkers with a variety of functionalized groups is electrografting. Electrografting is a technique that involves grafting functionalized organic linkers onto the electrode surface by applying a voltage to the working electrodes. The electrografting can be performed using a few electrochemical techniques, such as cyclic voltammetric (CV), and chronoamperometric techniques [54,55].

**Figure 11:**
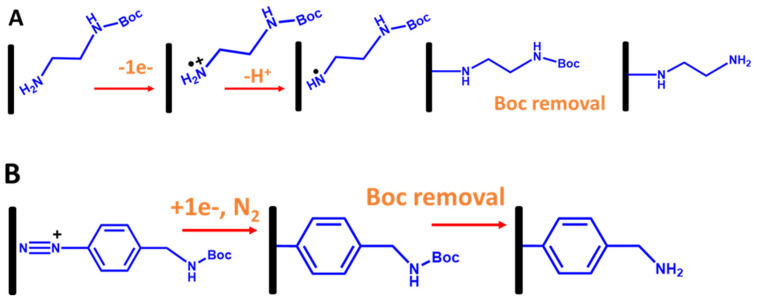
Schematic representation for stepwise electrografted primary amine and aryl linkers with Boc protecting groups onto the carbon electrode surfaces. (**A**) Electrografting of primary amine linkers via oxidation of amine group and (**B**) electrografting of aryl linkers via reduction in aryl diazonium salt [56].

In the case of immobilization ssDNA probes with electrografted alkyl linkers, primarily primary amine linkers are used. The most common method is electrooxidation of the amine group (the head group) onto the carbon surface using CV, as shown in Figure 11A, where the amine oxidation can be observed at a potential greater than 1.45 V vs. Ag/AgCI, as shown in Figure 11A [56,57]. However, a diamine linker gives unwanted effects, such as forming an amine bridge on the electrode surface. To tackle this problem, a protecting amine linker with a Boc protecting group (where Boc is tert-butyloxycarbony) is used in which the Boc group’s role is to inhibit the formation of an amine bridge. As established in Bartlett’s group [56,57], to electrograft amine linkers onto the electrode surfaces, Boc primary amine linkers were used, as shown in Figure 11B. After the electrografted Boc-amine linker was obtained, a Boc group removal was performed in 4 M HCl in a DMF solution to obtain electragrafted monolayer amine surfaces. Interestingly, the aminated surface-based primary amine linker can enhance the oxygen reduction reaction (ORR) in aerated solutions. This is due to the fact that the amine groups on the electrografted monolayer film provide electrocatalytic sites for ORR at the modified electrode [54].

**Figure 12:**
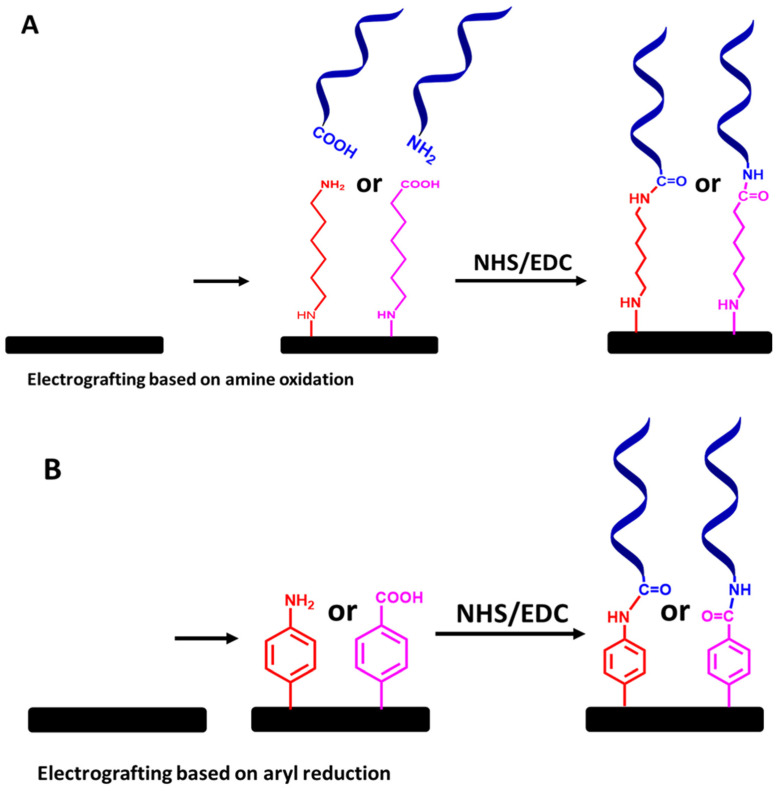
Step-by-step protocols for ssDNA molecular probe attachment using (**A**) electrografted primary amine linkers and (**B**) electrografted diazonium salt linkers, followed by NHS and EDC chemistry. The relative sizes of carbon electrodes, primary amine linkers, diazonium salt linkers, and ssDNA chains are not to scale.

The second most common approach is utilizing electrografted diazonium salt linkers with various functionalized group terminals. It is essentially through an electroreduction in aryl radicals of diazonium salt molecules, which subsequently bind to electrode surfaces, as shown in Figure 11B [58,59,60]. However, like electrografting of alkyl amine linkers, electrografting diazonium salts also need a few strategies. This is because the main problem is that electrografting of direct diazonium could also produce multilayers or polymeric layers due to the electroreduction in further aryl radicals at the 3′ and 5′ positions of the aryl group already attached on the electrode surface [61]. Therefore, to overcome this, multiple steps of electrografting diazonium chemistry are needed. Moreover, direct attachment to the diazonium functionalized amine is impossible due to a nucleophilic attack of the amine on the diazonium salts [58,59]. To tackle this issue, as established by Bartlett’s group, a diazonium salt linker with a Boc-protected amino group can also be used (Figure 12B). The Boc group plays a role in discouraging the formation of polymeric layers on the surface by blocking the coupling of other aryl groups at the 3 and 5 positions.

Finally, to create a covalently immobilized ssDNA probe on the electrode sensor for the detection of VRE genes via electrografted alkyl or diazonium salt linkers, functionalized ssDNA chains with amine or carboxy groups can be covalently linked to the electrografted linkers via of NHS and EDC chemistry, as illustrated in Figure 12A,B. Although this approach requires a tedious preparation step, the immobilized ssDNA molecular probes’ stability is the most essential aspect to consider.

### 3.3. Label-Free and Labeled Detection Systems

**Figure 13:**
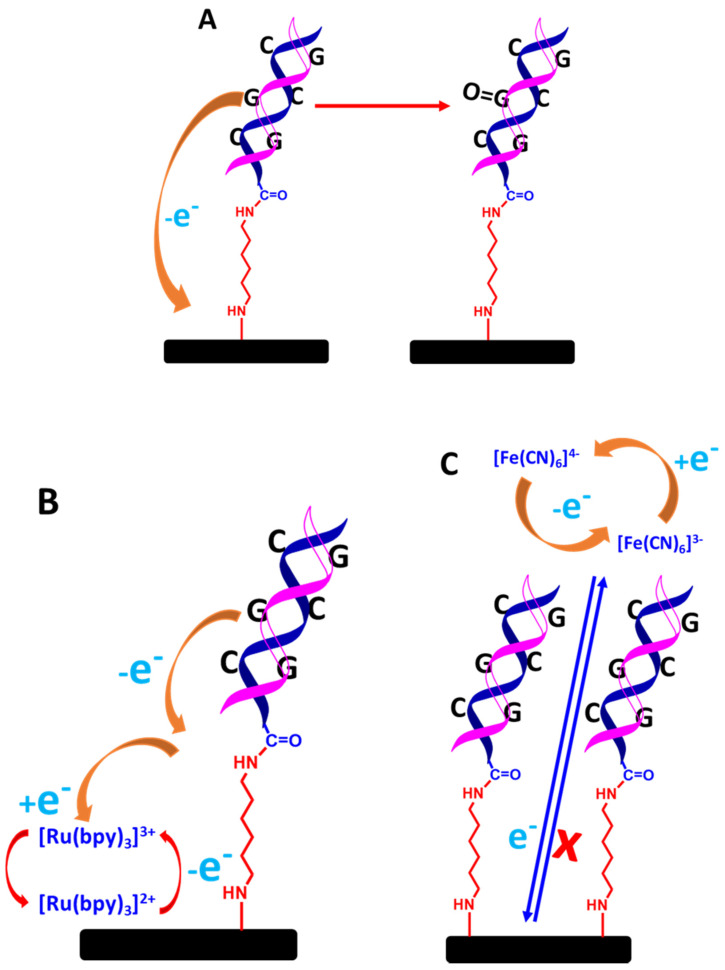
Schematic representation of detecting dsDNA based on label-free electrochemical biosensors; (**A**) guanine oxidation only, (**B**) guanine oxidation mediated by ruthenium complex in solution, and (**C**) blocking effect of ferrocyanide and ferricyanide redox couple [Fe(CN_6_)]^3−/4−^ in solution. The relative sizes of carbon electrodes, primary amine linkers, dsDNA chains are not to scale.

Two different types of DNA biosensing systems could be developed: label-free and labelled detection systems. In the case of the label-free system, two different approaches can be employed. First, the detection of VRE gene targets can be monitored through guanine oxidation, as shown in Figure 13A [62]. For the used guanine as a detection system, the oxidation of guanine after the formation of hybridized dsDNA can approximately be monitored between 0.9 and 1.0 V. As a result, the generated current decreases due to the reduced accessibility of guanine by the dsDNA structure, which inhibits the oxidation of guanine on the electrode surface [63,64]. In a different approach, redox mediator in a solution such as Tris (2,2′-bypridyl) dichlororuthenium(II) hexahydrate (Ru(bpy)^2+^) can be used, as illustrated in Figure 13B. This protocol enhances guanine oxidation [65]. Both approaches are simple, straightforward, rapid, and inexpensive to develop for the detection systems, but these methods require a more positive potential window to detect the guanine oxidation signal (>0.9 V). Moreover, the guanine oxidation signal can also be messed up by a false reading from the detection system caused by the non-specific adsorption of complementary DNA targets on the electrode surface that are not going through a hybridization reaction.

The second approach is monitoring the decreased current from the ferrocyanide or ferricyanide solution through voltammetric measurements. The decreased current is due to the blocking effect of the hybridized dsDNA structure towards [Fe(CN_6_)]^3−/4−^ electrochemistry on the electrode surface, as shown in Figure 13C [65]. It is also because [Fe(CN_6_)]^3−/4−^ and the negatively 3^−^/4^−^ charged phosphate of DNA strands strongly repel each other, which impedes [Fe(CN_6_)]^3−/4−^ electrochemistry even more. This method is also straightforward to implement; however, when employing voltammetric techniques such as CV, DPV, and SQW to detect a very small DNA target concentration, the electrochemical signals from the inhibition of [Fe(CN_6_)]^3−/4−^ are not that significant in terms of decreased current. Hence, to make a more sensitive measurement, EIS is the best electrochemical technique, as the EIS is very sensitive to monitor any changes on the electrode surface, particularly the formation of a double-helix DNA structure. As a result, the hybridized dsDNA structures significantly decrease the conductivity and increase the surface resistance of the modified electrode. In a label-free impedimetric measurement, a ferrocyanide and ferricyanide solution with an equimolar is used. The detection response is monitored through Nyquist plots, where the charge transfer resistance (*R*ct) can be determined. The increase in the semicircle diameter of the Nyquist plot is directly proportional to the *R*ct value. This signifies that the hybridized dsDNA has been formed on the electrode surface. Nevertheless, analyzing the data for the calibration plot will not be a straightforward analysis as the *R*ct value needs to reference the equivalent circuit model representing the electrical response of the electrochemical interface. Consequently, this approach requires skilled personnel who understand the physiochemical processes [Fe(CN_6_)]^3−/4−^ electrochemistry at the electrode/electrolyte interface.

**Figure 14:**
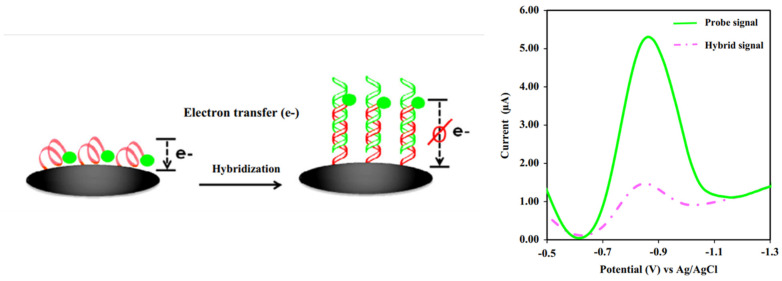
Schematic representation of detecting dsDNA based on a labeled system electrochemical DNA biosensor. This figure has been reproduced from Jampasa and co-workers [65].

In contrast, for a labeled system, the primary detection system is developed based on tagging an ssDNA probe using redox molecules. The frequent redox molecules used are ferrocene [66,67], methylene blue (MB) [68] blue, or anthraquinone (AQ) [52], where the molecules are tagged at the 3′ or 5′ of ssDNA chains. The key aspect of the detection system is developed via the generated voltammetric current from the redox reactions of the tagged redox moieties from ssDNA chains to the electrode surface. By forming the hybridized dsDNA at the electrode surface, the generated current from the tagged redox moieties reduces as the double helix DNA structure strongly inhibits the electron transfer of redox moieties [69]. Thus, it is more difficult for the electron(s) to transfer from the redox moieties, as depicted in Figure 14. As the concentration of DNA target increases, the number of dsDNA molecules on the electrode surface also increases, making it more difficult for the electron(s) to transfer to the electrode surface. This is strongly supported by the fundamental findings reported by Barton and co-workers [70], where the rate of electron transfer of MBs covalently tethered DNA in single-stranded strains is faster than the rate of electron transfer in double-stranded strains (as presented in Table 1 in the original paper). Thus, a significantly decreased voltammetric current is obtained. This method can be utilized to construct an assay for detecting DNA strains in the analytes of interest. Although many experimental reports on electrochemical DNA biosensing discussed how the voltammetric signals of the tagged redox molecules decrease after the hybridized dsDNA forms due to an increase in the distance of the redox molecules to the electrode surface, as discussed by Barton et al. [70], the activities of redox molecules strongly influence the redox signal intensity from the tagged redox moieties at DNA films, whether mediated by the DNA base pair stack or direct electron transfer from redox probes to the electrode surface. In addition, they also discussed the voltammogram intensity depending assembly conditions of ssDNA chains on the electrode surface and on the types of buffer solutions used during the assay. These variables significantly impact the elongation of dsDNA strains towards the electrode surface. Hence, this kind of behavior must be taken into account during the voltammetric measurements in order to achieve the highest overall sensitivity on the detection system [70]. Furthermore, the detection system is more sensitive if the sensing DNA interface is incorporated with very sensitive voltammetric techniques such as DPV and SWV in which both methods can give detection signals as low as 10^−9^ M. In addition, to accurately construct an analytical curve, it would be a great approach if the background current of DPV or SWV responses are subtracted from the voltammograms.

In a different approach, the electrochemical DNA sensors can be developed using various nanomaterials as modifiers on the electrode surfaces to enhance the detection systems. Commonly used nanomaterials in fabricating DNA biosensors include carbon nanomaterials (carbon nanotubes or graphene) and gold nanoparticles, via coated films that provide nanoplatforms from ssDNA molecule immobilization. The utilization of nanomaterials can increase the electroactive surface area [71]. Thus, more linkers can be electrografted onto the modified surfaces. As a result, more ssDNA molecules can covalently be attached to the electrode surface. In addition, the presence of nanomaterials can improve the conductivity of the modified electrode surfaces [72]. Both factors can give higher sensitive measurements in the detection of VRE genes.

## 4. Method of DNA Extraction of Real Samples for Electrochemical DNA Biosensors

**Figure 15:**
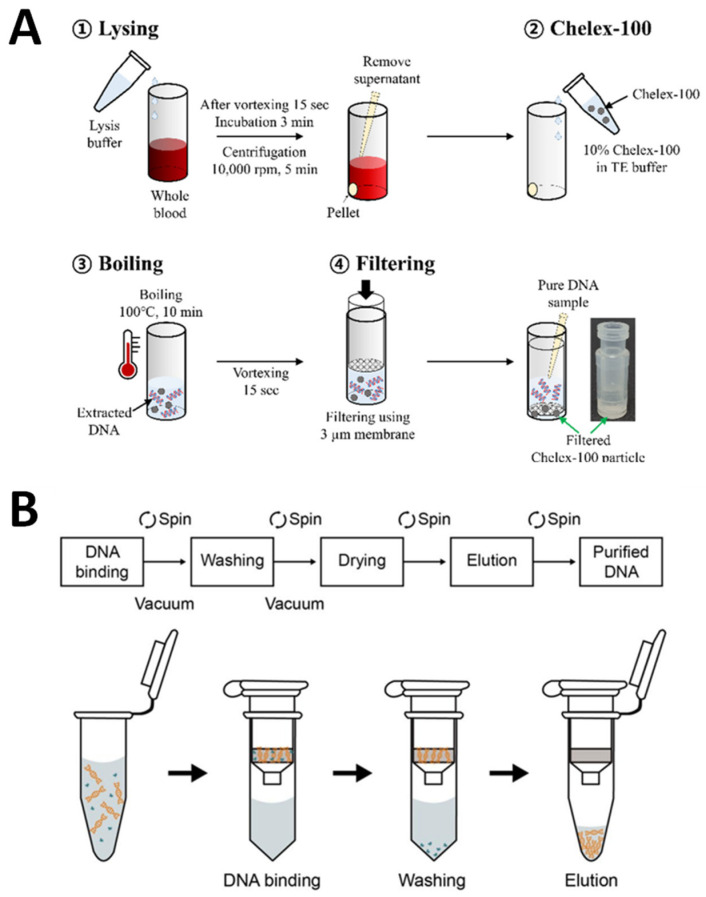
Schematic representation of DNA extraction for real sample measurements. (**A**) Conventional methods and (**B**) commercial DNA extraction kits. Subfigure (**A**) was adapted from Ref. [73], while subfigure (**B**) was retrieved from Ref. [74].

After the electrochemical DNA biosensors for VRE genes were successfully developed, a crucial part of our study was testing the developed DNA biosensors on real biological samples to confirm the VRE infections. Hence, the VRE genes were extracted from Gram-positive-bacteria in various real human samples such as infected wounds, serum, stool, and urine. Therefore, the extraction of DNA for real sample measurements is a vital process of this study. Figure 15A depicts DNA extraction from real samples using conventional methods, while Figure 15B demonstrates DNA extraction from real samples utilizing commercial DNA extraction kits.

A typical and common challenge confronted by biosensors developed for application to real samples is the matrix of the specimen samples, which may likely interfere with the results or negatively affect the biosensor’s detecting principle. Several considerations, such as the sample type, the amount of sample available, the cost of the extraction methods, and the time it takes to extract the DNA, influence the decision of which DNA extraction method to use. Liao and his group have described the difficulties involved with the sample preparation required for diagnostic testing for clinical specimens such as blood (whole blood, serum, or plasma), stool, urine, tissue, sputum, and saliva [75]. There are a few essential criteria for obtaining satisfactory accuracy during DNA protocol extraction: lysis, precipitation, and purification [76]. During lysis, the nucleus and cell are shattered by physical or chemical procedures such as centrifugation, sonication, grinding, and heating treatments with or without a detergent. In the following step, precipitation separates and eliminates nucleoprotein complexes and other impurities such as membrane lipids and nucleic acids. The last criterion is purification, which degrades biomolecules by employing digestive buffers or enzymes and obtains purified DNA only in a solution. Therefore, the methods that are most used for DNA extraction, which are conventional extraction and commercial kits, are discussed.

Conventional extraction methods, as in Figure 15A, such as the use of phenol-chloroform-isoamyl alcohol, are organic extraction processes widely applied in many laboratories for DNA extraction. These types of procedures involve first lysing cells to produce lysates and then inactivating cellular nucleases such as RNase. To remove the macromolecules, including protein, lipids, carbohydrates, and cell detritus, from the lysate, a combination of organic solvents, including phenol, chloroform, and isoamyl alcohol, is used. DNA is often precipitated using a solution of alcohol and salt after this process. Centrifugation is used to separate the DNA, and then, resuspensions are made by washing them in ethanol. These methods are costly, labor-intensive, and time-consuming when applied to a large amount of DNA [77]. Souvik et al. have reported a basic and novel method of collecting samples (from buccal swabs, urine, and hair) and extracting DNA using the phenol-chloroform method that was cost-effective, was easy to apply, and could be rapidly applied [78]. Moreover, many publications have thoroughly explored the toxicity and carcinogenicity of phenol and chloroform using this approach [79,80]. Additionally, low DNA yield and purity are the main drawbacks due to the use of detergent and residual cell debris that could contaminate the DNA samples and inhibit further applications.

As previously mentioned, the conventional method of extracting DNA has drawbacks; therefore, several strategies for rapid DNA extraction and more affordable methods have been reported. A variety of commercial DNA extraction kits used are shown in Figure 15B. The kits are extensively used in various DNA extractions of real samples [81,82]. This is because the commercially available kits can make use of the extraction of DNA, which enables the recovery of high levels of DNA purity and high yields. This is because they can work with robotic platforms and have limited hands-on time for conducting major experiments [83]. Commercial DNA extraction kits are standard in extracting DNA from cells, as the first step is lysing the cells in an alkaline solution. Then, these processes are followed by incubating the mixture with a DNA-binding matrix, washing the mixture, and finally eluting the DNA. The spin columns in commercial kits include a silica resin that binds DNA selectively, depending on the salt conditions and other parameters that affect the extraction procedure. Ali and co-workers have studied and compared the modifications of a phenol/chloroform extraction method plus an inhibitor-removal solution (C3) (ph/Chl + C3) to the PowerFecal^®^ DNA Isolation Kit (MoBio-K). Although the Ph/Chl + C3 approach took less time, there were some health and safety problems with phenol and chloroform exposure and disposal compared to MoBio-K, which is simple and straightforwardly applied on the benchtop with standard laboratory safety measures [84].

It is essential to extract DNA in high quantities and quality to improve the DNA extraction method. DNA extraction using magnetic nanoparticles (MNPs) is widely recognized as a powerful technique. MNPs have a high surface-area-to-volume ratio, a high binding rate with detecting compounds, and the ability to perform magnetically regulated aggregation and dispersion. Because of their high dispersibility, MNPs are also able to rapidly and efficiently bind to biomolecules. Chen et al. studied MNPs and used them to develop an automatic quick nucleic acid extractor that can process 16 samples at the same time. The nucleic acid extraction method can perform an entire extraction in 30 min and is reliable and stable [85]. A magnetic field draws the target-bound molecule towards the magnet, separating it from undesired material or inhibitors without disrupting the target nucleic acid. The extraction of nucleic acids using magnetic beads has become routine in current molecular biology.

In summary, the optimal DNA extraction method of real samples for electrochemical DNA biosensors should meet the following criteria: being simple, sensitive, rapid, and efficient. DNA extraction techniques should also take the following into consideration: high DNA recoveries, impurity and inhibitor removals, and high-throughput processing.

## 5. Future Outlook

Conventional detection methods for VRE genes have been commonly used in hospital clinical laboratories. However, the detection costs are still too high, especially in low- and medium-income countries. Consequently, reducing detection costs and simplifying detection systems have always been the primary goals in industrial and commercialization detection. The development of electrochemical DNA biosensors can replace one of the most traditional methods since interest in bacteria detection biosensing technology-based electrochemical measurements is rising. Conventional gold (Au) and glassy carbon (GC) electrodes are commonly utilized in developing electrochemical DNA biosensors for bacterial detection in which a three-electrode system (working, counter, and reference electrodes) is manually set up. Nevertheless, when using screen-printed electrodes (SPEs), such as screen-printed carbon electrodes (SPCEs) and screen-printed gold electrodes (SPGEs), measurements are easy because the three-electrode system is on the same electrode platform. This makes them an attractive disposable sensor for detecting VRE genes. Screen-printed electrodes offer numerous advantages, including low cost, design flexibility, high reproducibility, microvolumes of samples used, and a wide range of options for modifying the properties of electrode surfaces. During hospital outbreaks, it is important and necessary to reduce the time needed to diagnose VRE. Thus, using electrochemical DNA biosensors to detect VRE genes-based SPEs have a significant potential for making high-selectivity detection systems by creating DNA platforms that can only detect sequence-specific activity towards the VRE genes of interest. Additionally, the modified electrodes allow for rapid in situ analysis with high reproducibility, sensitivity, and accuracy.

Recent advances in 3D printing technology and the availability of many conductive thermoplastic filaments to produce 3D-printed electrodes have enabled the additive manufacturing of 3D biosensing devices. Typically, this is accomplished by printing the electrodes with various conductive thermoplastic carbon nanomaterials, which are a composite of the polylactic acid (PLA) polymer and carbon nanomaterials (graphene, carbon nanotubes, and carbon black) with a range of designs and specified geometries [86]. However, the most crucial consideration is how to immobilize the ssDNA probe on the printed electrode substrate. Consequently, the printed electrodes can be evaluated as an electrochemical DNA biosensor capable of detecting VRE genes.

Even though DNA biosensors are often utilized in electroanalysis, extensive data display platforms are still required for signal output. This flaw not only raises detection costs but also affects the growth of on-site detection. Due to the widespread use of smartphones, many studies have developed applications that generate detection signals. The smartphone’s high-quality camera, portability, and feasibility make it a great development candidate. Additionally, smartphones can be used as reading devices. It may become a research focus in the future for the development of miniaturized and portable electrochemical DNA biosensors for detecting VRE genes. Table 2 summarizes the advantages and disadvantages of the essential methods for detecting VRE genes.

## Figures and Tables

**Table 2:** Summary of the advantages and disadvantages of detecting VRE.

Diagnostic Method	Advantages	Disadvantages	Preparation and Detection Times	References
Conventional PCR	More cost effective than culture and staining	Lengthy analysis, sterile setting, no on-site testing	Less than 1 days	[42,87]
Multiplex PCR	Combined amplification of many gene types	Primer annealing temperatures	Less than 1 days	[39,40]
RT-PCR	Detection of living cells with high purity and specificity	Instability of the RNA molecule	Less than 1 days	[14,28,43]
ELISA	High specificity, user-friendliness, quantitative, and qualitative	The unstable, high false-positive rate	Less than 1 days	[31]
Immunoassay methods	Portable, disposable, and with a lower detection limit than conventional immunological methods	Batch-to-batch (or clone-to-clone) variability and antibody instability	2–3 days (detection in 5–7 min)	[32]
Electrochemical DNA biosensors	Real-time detection, high sensitivity and specificity, and low cost and can be miniaturized.	Sample preparation is dependent on a bioreceptor.	4–10 h (detection in 5 min)	[88,89,90]
